# Alternating Current-Dielectrophoresis Collection and Chaining of Phytoplankton on Chip: Comparison of Individual Species and Artificial Communities

**DOI:** 10.3390/bios7010004

**Published:** 2017-01-05

**Authors:** Coralie Siebman, Orlin D. Velev, Vera I. Slaveykova

**Affiliations:** 1 Environmental Biogeochemistry and Ecotoxicology, Department F.-A. Forel for Environmental and Aquatic Sciences, Earth and Environmental Science, Faculty of Sciences, University of Geneva, 66 Boulevard Carl-Vogt, CH-1211 Genève 4, Switzerland; coralie.suscillon@unige.ch; 2 Department of Chemical and Biomolecular Engineering, North Carolina State University, Raleigh, NC 27695-7905, USA; odvelev@ncsu.edu

**Keywords:** AC dielectrophoresis, chaining efficiency, green algae, cyanobacteria, diatom, phytoplankton, freshwater

## Abstract

The capability of alternating current (AC) dielectrophoresis (DEP) for on-chip capture and chaining of the three species representative of freshwater phytoplankton was evaluated. The effects of the AC field intensity, frequency and duration on the chaining efficiency and chain lengths of green alga *Chlamydomonas reinhardtii*, cyanobacterium *Synechocystis* sp. and diatom *Cyclotella meneghiniana* were characterized systematically. *C. reinhardtii* showed an increase of the chaining efficiency from 100 Hz to 500 kHz at all field intensities; *C. meneghiniana* presented a decrease of chaining efficiency from 100 Hz to 1 kHz followed by a significant increase from 1 kHz to 500 kHz, while *Synechocystis* sp. exhibited low chaining tendency at all frequencies and all field intensities. The experimentally-determined DEP response and cell alignment of each microorganism were in agreement with their effective polarizability. Mixtures of cells in equal proportion or 10-times excess of *Synechocystis* sp. showed important differences in terms of chaining efficiency and length of the chains compared with the results obtained when the cells were alone in suspension. While a constant degree of chaining was observed with the mixture of *C. reinhardtii* and *C. meneghiniana*, the presence of *Synechocystis* sp. in each mixture suppressed the formation of chains for the two other phytoplankton species. All of these results prove the potential of DEP to discriminate different phytoplankton species depending on their effective polarizability and to enable their manipulation, such as specific collection or separation in freshwater.

## 1. Introduction

The phytoplankton in aquatic systems are highly complex and heterogeneous. Phytoplankton includes an assembly of diverse photoautotrophic species, such as eukaryotic algae, diatoms and cyanobacteria developing in the euphotic zone of natural waters [1,2]. They have an important ecological role, being central for primary productivity in surface water, as well as being essential food sources for zooplankton, fish and mammals [1,2]. Because of their capability to rapidly respond to environmental changes due to their small size and fast metabolic processes, phytoplankton are considered good indicators of water quality [3,4]. In recent years, phytoplankton cells were widely used as biological components in biosensors for water monitoring and demonstrated their sensitivity to a large range of aquatic pollutants, including herbicides, pesticides and toxic metals [5,6,7]. Phytoplankton from the aquatic environment combined with dielectrophoresis (DEP) could offer new means for the development of biosensors for water quality assessment. Previous studies showed the capability of DEP to electrically control the trapping and focusing of bioparticles [8,9,10]. However, despite the recent introduction of DEP-based devices using bioparticles for water quality assessment [11,12,13,14,15], only a few studies have focused on DEP manipulation of live cells and their mixtures [15], and no studies have explored the DEP behavior of representative species from the phytoplankton apart for the green microalga *Chlamydomonas reinhardtii* [16]. The DEP response of freshwater microorganisms in natural water is thus largely unexplored, and its potential as a tool for the manipulation of complex cell systems in realistic environments has not been fully assessed. Alternating current (AC)-DEP-driven collection and chaining of cells or particles is also an important process in the operation of whole cell biosensors based on 2D arrays of cells [17], as the chaining process represents the first step in the formation of a 2D array [18,19].

The formation of one-dimensional arrays or “pearl-chains” in the direction of the applied electric field involves cell-cell DEP chaining force *F_c**hain**_* [8,19,20,21,22]:

(1) $$F_{chain}=-\;C\pi \varepsilon_{m}Re{\left|CM\left(\omega \right)\right|}^{2}r^{2}E^{2}$$

where $\varepsilon_{m}$ is the permittivity of the surrounding media, *r* is the radius of the cell, *E* is the electric field intensity, the coefficient *C* depends on the number and distance between the cells within the growing chain (3 < *C* < 10^3^) and CM(ω) is the Clausius–Mossotti factor, whose real part *Re*|| corresponds to the polarizability function of a bioparticle and depends on the complex permittivity of the medium $\varepsilon_{m}^{*}$ and the cell $\varepsilon_{cell}^{*}$ [8,19,23] (Equation (2)):

(2) $$CM\left(\omega \right)=\frac{\varepsilon_{cell}^{*}-\varepsilon_{m}^{*}}{\varepsilon_{cell}^{*}+2\varepsilon_{m}^{*}}$$

Examples of microorganisms captured in one-dimensional arrays include viable yeast using castellated microelectrodes [21], coplanar gold electrodes [19,24] or interdigitated electrodes [25], different strains of bacteria using curved microelectrodes [26], polynomial and castellated electrodes [27] or interdigitated electrodes [15,28], as well as microalgae with coplanar gold electrodes [16] and protozoan parasite with interdigitated electrodes [15]. However, the chaining process of phytoplankton cells under realistic conditions, such as in complex mixtures of representative phytoplankton cells, has not been assessed yet despite its high environmental relevance.

The major goal of the present study is to understand the DEP behavior of representative phytoplankton species and to determine to what extent and under what conditions the mixture of these phytoplankton cells could affect the dielectrophoretic response of individual cells and thus influence the phytoplankton cell trapping and chaining in microfluidic on-chip devices. Green microalga *C. reinhardtii*, cyanobacterium *Synechocystis* sp. and diatom *Cyclotella meneghiniana* were chosen as representative model organisms for the freshwater phytoplankton. These three phytoplankton species were hypothesized to show differing DEP behaviors under specific AC field intensities and frequencies due to the differences in their size and cell wall composition, which are expected to affect their DEP behavior, allowing their separation when mixed in suspension. Indeed, *Synechocystis* sp. has a size 5 times smaller than *C. reinhardtii* or *C. meneghiniana*, while *C. meneghiniana* was chosen because of the specific composition of the cell wall, including silicate, compared to *C. reinhardtii* and *Synechocystis* sp. The influence on the chaining efficiency of the AC field intensity, frequency and duration for individual phytoplankton species and their mixtures was explored.

## 2. Materials and Methods

### 2.1. Cell Cultures and Test Medium

*C. reinhardtii* (CPCC 11, Canadian Phycological Culture Centre, Waterloo, ON, Canada) and *C. meneghiniana* (1020-1a, Experimental Phycology and Culture Collection of Algae at the University of Goettingen, Goettingen, Germany) were cultured at 20 °C under rotary shaking at 115 rpm and continuous illumination of 6000 lux (INFORS HT, Basel, Switzerland) in a four-times diluted Tris-acetate-phosphate medium (Sigma-Aldrich, Buchs, Switzerland) and Talaquil medium, respectively. *Synechocystis* sp. (PCC 6803, Canadian Phycological Culture Centre, Waterloo, ON, Canada) was grown under the same temperature and shaking conditions, but under day-night illumination of 6000 lux (INFORS HT, Basel, Switzerland) in BG-11 Blue­Green medium. The cells were collected at the mid-exponential growth phase and isolated from each growth medium by centrifugation at 3000 rpm for 10 min (Omnifuge 2.0 RS, Heraeus Sepatech GmbH, Osterode/Harz, Germany). The supernatant was removed, and the cells were re-suspended in Geneva Lake water with physico-chemical composition detailed in Table S1 of the Supplementary Materials and filtered through 0.45-µm pore size filters (Millipore, Billerica, MA, USA). The final cell concentration was 5 × 10^6^ cells·mL^−1^ for *C. reinhardtii* and *C. meneghiniana* and of 5 × 10^7^ cells·mL^−1^ for *Synechocystis* sp. (if not specified otherwise).

### 2.2. DEP Experimental Setup and Parameter Optimization

DEP assembly experiments were performed with coplanar gold electrodes separated by a 2-mm gap enclosed in a 350-μm thick transparent microfluidic chamber as described elsewhere [16,24,29] and chosen based on several advantages, including their simple and robust use [16,19]. The gold electrodes were vapor-deposited onto 25 × 75 mm microscope glass slides. The fabrication and the electrical actuation of these electrodes were the same as previously described [16]. The applied frequencies were limited to a maximum of 500 kHz to avoid distortion of the AC signal created by the amplifier connected to the coplanar electrodes.

### 2.3. DEP Behavior of Representative Phytoplankton Species

DEP behavior was explored for each phytoplankton species individually and in mixtures. Experiments were performed to understand the DEP behavior of representative phytoplankton species. The electrical field intensity, frequency and duration, as well as the phytoplankton concentration were systematically varied for each phytoplankton species to find the optimal combination of these parameters allowing the formation of the cell chains. AC field intensities of 15 V·mm^−1^, 20 V·mm^−1^ and 25 V·mm^−1^ for frequencies increasing from 100 Hz to 500 kHz were tested. The effect of AC field duration from 5 min to 30 min on the chaining formation was also explored.

To explore the DEP behaviors of phytoplankton under environmentally-realistic conditions, mixtures of these phytoplankton species forming artificial communities were also tested; first, binary mixtures containing equal proportions of cells (1:1): *C. reinhardtii* + *C. meneghiniana*, *C. reinhardtii* + *Synechocystis* sp. and *C. meneghiniana* + *Synechocystis* sp. Cell concentrations were kept at 2.5 × 10^6^ cells·mL^−1^. Then, mixtures containing *C. reinhardtii* or *C. meneghiniana* and 10-times excess of *Synechocystis* sp. were tested: the concentration of *C. reinhardtii* and *C. meneghiniana* was fixed at 2.5 × 10^6^ cells·mL^−1^, while the concentration of *Synechocystis* sp. was fixed at 2.5 × 10^7^ cells·mL^−1^. For all experiments with cell mixtures, 2 AC field intensities of 15 and 25 V·mm^−1^ and 3 frequencies of 100 Hz, 1 kHz and 500 kHz were applied for 5 min. Each experiment was repeated 3 times.

### 2.4. Effect of AC Field on Membrane Permeability of the Cells

The possible effect of the AC field on the membrane permeability of the cells was investigated by flow cytometry (FCM) using propidium iodide (PI, Life Technologies Europe B.V, Zug, Switzerland) stain. FCM analyses were performed using a BD Accuri C6 flow cytometer (BD Biosciences, San Jose, CA, USA) with an Accuri CSampler (BD Biosciences, San Jose, CA, USA). The number of cells and PI fluorescence emitted at 585 nm after excitation with a 488-nm argon laser were acquired and analyzed using the BD Accuri C6 Software 264.15 (BD Biosciences, San Jose, CA, USA). Data were collected to 10,000 events for each sample. *C. reinhardtii* and *C. meneghiniana* treated with 50 µM hydrogen peroxide (Life Technologies Europe B.V, Zug, Switzerland) were used as positive controls, whereas for *Synechocystis* sp., the cell suspension was heated at 70 °C for 20 min. For all phytoplankton species, the negative control corresponds to the cell suspension with no AC field treatment. PI at 7 µM was added and incubated for 30 min in negative and positive controls, as well as in all samples prior to FCM analysis.

### 2.5. Microscopy and Image Analysis

The DEP behavior of the cells in the microfluidic chamber was observed at the bottom of the microfluidic channel, in the center between the electrodes on an area of approximately 250 μm × 250 μm with an optical microscope (BX61, OLYMPUS, Volketswil, Switzerland) using a digital camera (XC30, OLYMPUS, Volketswil, Switzerland) and the Cellsens software (Cellsens dimension OLYMPUS, Volketswil, Switzerland) provided. For each combination of tested parameters (field intensity, frequency, duration, concentration of phytoplankton and mixtures of phytoplankton), microscopic images were collected every 5 min for 30 min after AC field application.

### 2.6. Cell Chaining Efficiency Determination

The efficiency of chain formation was characterized by the percentage of cells in chains and the length of the chains determined using the image processing program ImageJ (National Institute of Mental Health, Bethesda, MD, USA). The percentage of cells in chains was calculated for each image according to:

(3) $$Cells\;in\;chains\;\left(\%\right)=\frac{\sum number\;of\;chains\times number\;of\;cells\;per\;chain}{total\;number\;of\;cells\;in\;the\;system}\times 100$$

For each condition, three replicate measurements were performed. The mean of the percentage of cells in the chain and the standard deviation were calculated and compared. In addition, the length of the chains was also determined. For each condition, the fraction of cells forming chains in the range of [0 to 5], [6 to 10], [11 to 15], [16 to 20], [21 to 25], [26 to 30] and [>30] cells per chains are reported as follows:

(4) $$Fraction\;of\;\mathrm{cells}\;\left(\%\right)=\frac{number\;of\;cells\;in\;chains\;in\;\left[x\;to\;y\right]\;range}{total\;number\;of\;cells\;in\;chains}\times 100$$

### 2.7. Measurements of Cell Size and Zeta Potential

To calculate the effective polarizability of *C. reinhardtii*, *C. meneghiniana* and *Synechocystis* sp., the values of the zeta-potential and cell size in Geneva Lake water of each phytoplankton species are needed. Measurements of phytoplankton hydrodynamic size and zeta-potential were carried out by a Zetasizer Nano-ZS (Malvern, Renens, Switzerland). Three replicates of 7 measurements each were performed for both phytoplankton species, and the means are reported in Table 1.

### 2.8. Modelling of Chaining Efficiency

The chaining efficiency of the different phytoplankton species was modeled by using the common multishell model [19]. Numerical parameters used to determine the real part of the Clausius–Mossotti factor (Equation (2)) are listed in Table 1. The parameters used to calculate the effective polarizability for *C. reinhardtii* were assumed to be equivalent to those of *Chlorella vulgaris* [30] and those for *Synechocystis* sp. to *Escherichia coli* [31]. Since no data were available for *C. meneghiniana*, *C. reinhardtii* parameters were taken except for the numerical parameters related to the cell wall. Cell wall-related parameters for *C. meneghiniana* were approximated by the values known for SiO_2_ [32], since its frustule is made by silica [33].

For multishell particles, such as the green microalgae, bacteria or diatoms used in this study, complex cell permittivity is given by [8,9]:

(5) $$\varepsilon_{cell}^{*}=\varepsilon_{wall}^{*}\left[\frac{{\left(\frac{R_{0}}{R}\right)}^{3}+2\left(\frac{\varepsilon_{cyt}^{*}-\varepsilon_{wall}^{*}}{\varepsilon_{cyt}^{*}+2\varepsilon_{wall}^{*}}\right)}{{\left(\frac{R_{0}}{R}\right)}^{3}-\left(\frac{\varepsilon_{cyt}^{*}-\varepsilon_{wall}^{*}}{\varepsilon_{cyt}^{*}+2\varepsilon_{wall}^{*}}\right)}\right]+\varepsilon_{EDL}$$

where $\varepsilon_{wall}^{*}$ is the complex permittivity of the cell wall, $\varepsilon_{cyt}^{*}$ is the effective complex permittivity of the cytoplasm, $\varepsilon_{EDL}^{*}$ is the counter-ionic layer dielectric constant and *R**_o_*** and *R* are the outer radius and the inner radius of the cell [8,20]. The complex permittivity for the different shells was expressed as follows [8,19]:

Cytoplasm:

(6) $$\varepsilon_{cyt}^{*}=\varepsilon_{cyt}-\frac{j}{w}\sigma_{cyt}$$

Cell membrane:

(7) $$c_{mem}^{*}=c_{mem}-\frac{j}{w}g_{mem}$$

Cell wall:

(8) $$\varepsilon_{wall}^{*}=\varepsilon_{wall}-\frac{j}{w}\sigma_{wall}$$

Counter-ionic layer:

(9) $$\varepsilon_{EDL}^{*}=\varepsilon_{EDL}-\frac{j}{w}\sigma_{EDL}$$

where:

(10) $$\sigma_{EDL}=2\sigma_{m}R_{o}^{-1}k^{-1}e^{\left(\frac{ze\xi }{2kT}-1\right)}$$

with medium permittivity:

(11) $$\varepsilon_{m}^{*}=\varepsilon_{m}-\frac{j}{w}\sigma_{m}$$

### 2.9. Statistical Analysis

Statistical differences of chaining efficiency under different AC field treatments were determined using one-way ANOVA, the Student–Neumann–Keuls method for all pairwise multiple comparisons and performed in Sigma Plot 11 (Systat Software Inc., San Jose, CA, USA). The statistically significant differences (*p* < 0.05) are indicated by different letters.

## 3. Results

### 3.1. Collection and “Pearl” Chain Formation by the Individual Microorganisms

To understand the DEP phenotypes of individual cells representative of the phytoplankton, the chaining efficiency and chain length were explored at different AC field intensities and frequencies, durations, as well as different cell concentrations.

#### 3.1.1. Effect of AC Field Frequency and Intensity on Chaining Efficiency and Chain Length

**Figure 1:**
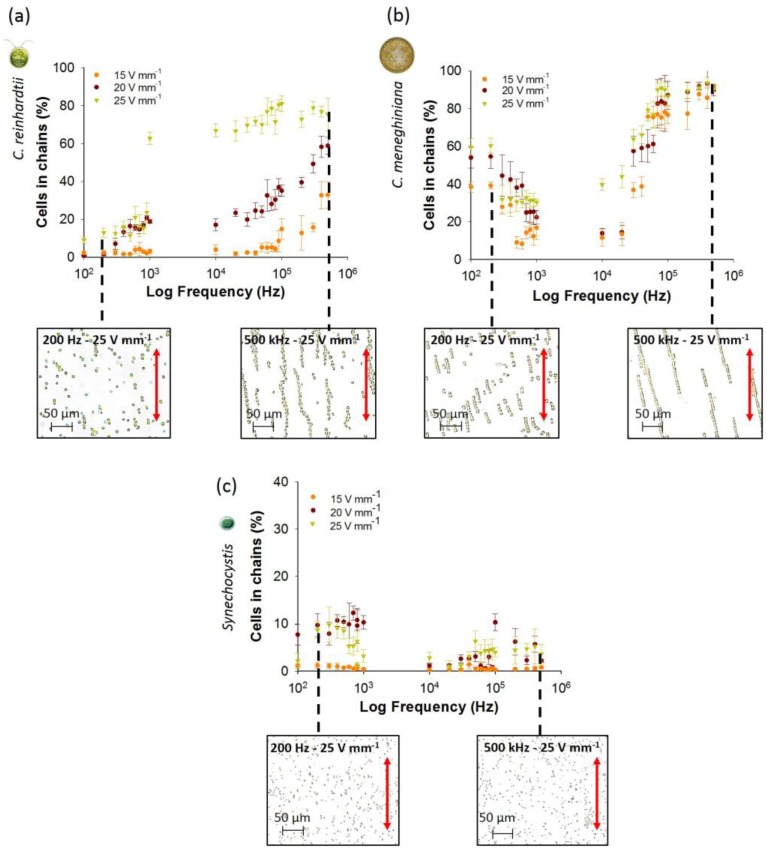
Effect of AC field intensity and low frequency on the chaining efficiency obtained with the coplanar electrode after 5 min of electric field application for: (**a**) *C. reinhardtii*; (**b**) *C. meneghiniana*; (**c**) *Synechocystis* sp. Micrographs illustrate the chaining pattern, parallel to the electric field direction given by the red arrow, for each phytoplankton species at 200 Hz and 500 kHz, field intensity of 25 V·mm^−1^. Initial concentration of phytoplankton species: 5 × 10^6^ cells·mL^−1^ *C. reinhardtii* and *C. meneghiniana*; 5 × 10^7^ cells·mL^−1^ *Synechocystis* sp.

For low frequencies from 100 Hz to 1 kHz, no change in chaining efficiency, as the percentage of cells in chains, was observed for *C. reinhardtii* at 15 V·mm^−1^ with increasing frequency (around 3% at 100 Hz and 1 kHz), while an increase of the chaining efficiency was obtained from 0.8% ± 0.2% and 9.1% ± 1.9% at 100 Hz to 18.8% ± 1.0% and 62.7% ± 3.2% at 1 kHz at 20 and 25 V·mm^−1^, respectively (Figure 1a).

For diatom *C. meneghiniana*, a decrease of the chaining efficiency was observed with the increase of the frequency from 100 Hz to 1 kHz for the three AC field intensities (from 59.7% ± 4.4% at 100 Hz to 30.4% ± 1.8% at 1 kHz and 25 V·mm^−1^) (Figure 1b). *Synechocystis* sp. showed lower chaining efficiency than diatom and green alga at all AC field intensities and frequencies (Figure 1c). At frequencies below 900 Hz and AC field intensities higher than 20 V·mm^−1^, the highest chaining efficiency was observed for *C. meneghiniana* as compared with *C. reinhardtii* and *Synechocystis* sp. (Figure 1). When comparing the chaining behavior of *C. meneghiniana* and *C. reinhardtii* at low frequencies, the opposite trend was found. Increasing the frequencies from 100 Hz to 1 kHz induced an increase of the chaining efficiency in *C. reinhardtii* and a decrease in *C. meneghiniana*. This phenomenon of high chaining efficiency at very low frequency of 100 Hz for *C. meneghiniana* could be caused by augmentation of the particle collection by fluid AC electroosmosis (ACEO). The ACEO is a complex phenomenon of coupling field gradients and fluid fluxes, where the gradient of the applied electric field between the electrodes induces a fluid motion away from the electrodes, which drags and concentrates the cells in the middle of the experimental chamber [34], as reported earlier with latex particles at low electric field frequencies [20,35]. It is likely that it enhances the chaining, and the larger cell type (*C. meneghiniana*) will be more susceptible to such fluid drag.

During the characterization of chaining at higher frequencies (Figure 1) for *C. reinhardtii*, an augmentation of the chaining efficiency was obtained by increasing the frequencies from 10 kHz to 500 kHz and the AC field intensities from 15 to 25 V·mm^−1^. A maximum of around 80% of cells in chains was reached from 50 kHz to 500 kHz at 25 V·mm^−1^, while lower chaining efficiency was reached at lower AC field intensities of 15 and 25 V·mm^−1^ (Figure 1a). These results are consistent with data reported earlier for yeast cells and bacteria [9,11,19,34], where an increase of the cell collection was observed at higher AC field intensity. For example, the increase of the capture efficiency of *E. coli*, *Salmonella* and *Pseudomonas* sp. from 90% to 99% was observed by increasing the AC field intensity from 67 V·cm^−1^ to 84 V·cm^−1^ [11]. Similarly, an increase from 50 to 200 V·cm^−1^ of the electric field intensity enhanced the chain length of yeast cells at all tested frequencies [19]. Unlike *C. reinhardtii*, *C. meneghiniana* showed no significant differences in chaining efficiency between the three tested AC field intensities at all frequencies (Figure 1b). An increase of the percentage of cells in chains occurred by increasing the frequency from 10 kHz to 100 kHz to reach a plateau of 90% of cells in chains from frequencies above 100 kHz. Chaining efficiencies for *Synechocystis* sp. were below 5% at all AC field intensities and frequencies from 10 kHz to 500 kHz (Figure 1c), as observed at low frequencies.

**Figure 2:**
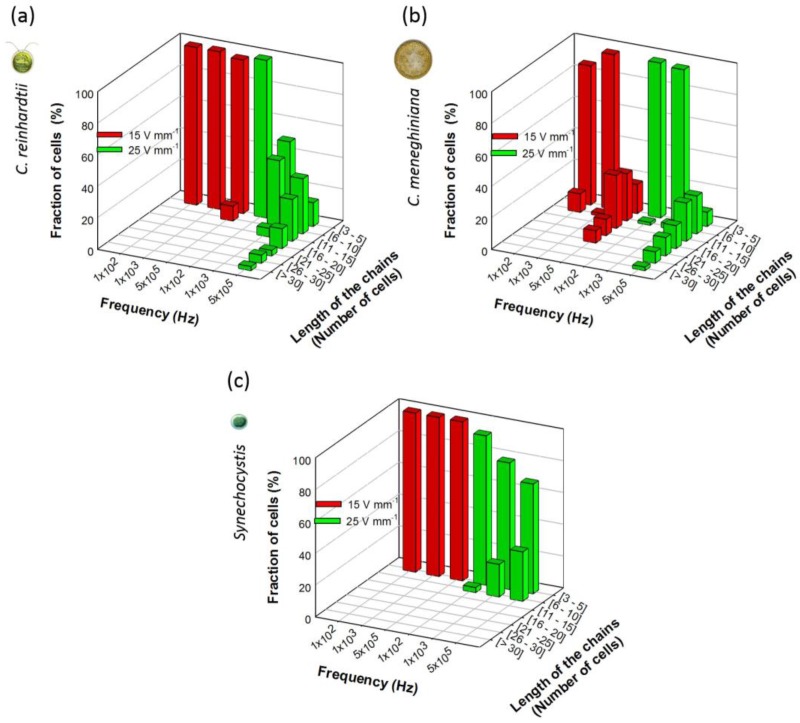
Length of the chains obtained at 15 and 25 V·mm^−1^ for 100 Hz, 1 kHz and 500 kHz for: (**a**) *C. reinhardtii*; (**b**) *C. meneghiniana*; and (**c**) *Synechocystis* sp. *C. reinhardtii* and *C. meneghiniana* initial concentrations: 5 × 10^6^ cells·mL^−1^; *Synechocystis* sp.: 5 × 10^7^ cells·mL^−1^.

The number of cells in chains was also compared to outline the differences in the distribution of the chain lengths of all three phytoplankton species for a field intensity of 15 and 25 V·mm^−1^ and frequencies of 100 Hz, 1 kHz and 500 kHz (Figure 2). *C. reinhardtii* formed long chains of more than 30 cells only at 500 kHz and 25 V·mm^−1^. At all other AC field intensity/frequency combinations, *C. reinhardtii* chains contained maximum 10 cells per chain (Figure 2a). At the highest frequency of 500 kHz, *C. meneghiniana* formed chains of 20 cells per chain at 15 V·mm^−1^ and chains of 30 cells and more at 25 V·mm^−1^ (Figure 2b), while chains of less than five cells per chain were observed at low frequencies for both field intensities. *C. meneghiniana* formed the longest chains, as they also showed the highest chaining efficiency as the percentage of cells in chains. By contrast, *Synechocystis* sp. formed short chains no longer than five to 10 cells at different AC field intensities and frequencies (Figure 2c). Taken together, the results demonstrate that the chaining efficiency and the length of the chains in freshwater phytoplankton decreased in the order: *C. meneghiniana* > *C. reinhardtii* >> *Synechocystis* sp. As both *C. reinhardtii* and *C. meneghiniana* exhibited the highest chaining efficiency and longer chain formation at high frequency and high AC field intensity of 500 kHz and 25 V·mm^−1^, this combination of AC field intensity and frequency was used in the further experiments to follow the effect of the cell concentration and the duration of the AC field on the chaining efficiency, as well as on the cell viability.

The obtained chaining efficiency results were consistent with the effective polarizability calculated with the multishell model. Negative values of −0.5 and −0.4 for *Re|CM(ω)|* of *C. meneghiniana* and *C. reinhardtii* and a positive value of 0.27 for *Synechocystis* sp. were determined from 100 Hz to 500 kHz. The obtained values were in accordance with the literature showing the numerical limits of the real part of the Clausius–Mossotti factor: −0.5 < *Re|CM(ω)|* < 1, independently of the particles’ properties [36]. These results imply that *C. meneghiniana* and *C. reinhardtii* might undergo negative DEP as opposed to positive DEP for *Synechocystis* sp. Indeed, several studies demonstrated the relation between the sign of *Re|CM(ω)|* and the sign of the DEP [37,38,39]. Positive DEP corresponding to the attraction of the particles to the maxima field regions is observed when *Re|CM(ω)|* > 0, while negative DEP to the repulsion of the particles from the high-field region is observed when *Re|CM(ω)|* < 0. For example, calculation of *Re|CM(ω)|* of *E. coli* predicted positive values of this factor and, thus, positive dielectrophoresis, p-DEP, at frequencies from 30 kHz to 100 MHz, which was confirmed by the attraction of the bacteria towards the regions of high field strength at the same frequencies [38]. Similarly, calculations of the effective polarizability of latex particles showed positive *Re|CM(ω)|* at frequencies below 10^6^ Hz, while negative *Re|CM(ω)|* at frequencies higher than 10^6^ Hz [38]. In experimental observations, low frequencies (down to 10 kHz) induced p-DEP to the particles, while negative DEP, n-DEP, was experienced at high frequencies (up to 20 MHz) [39]. Both can lead to chaining in the direction of the field [35]. Theoretical calculations and experimental observations in the present study were also consistent with those for protozoa *Cryptosporidium*. Indeed, the previously estimated *Re|CM(ω)|* of *Cryptosporidium* changed from positive to negative values at 3 MHz, which was confirmed with experiments where the protozoa showed p-DEP from 100 kHz to 1 MHz and n-DEP above 4 MHz [37].

The differences in *Re|CM(ω)|* values could therefore explain the difference between the high chaining efficiency of *C. meneghiniana* and *C. reinhardtii* and the low chaining efficiency of *Synechocystis* sp. Indeed the capability of cells to form chains seemed to be clearly correlated to their effective polarizability, as shown previously [18,19,35,38,40].

#### 3.1.2. Effect of Cell Concentrations on Chaining Efficiency

For all of the tested microorganisms, low chaining efficiencies were found at cell concentrations of 10^6^ cell·mL^−1^ (Figure S1): less than 40% of cells of *C. reinhardtii* and *C. meneghiniana* were in chains, while no chain formation was observed for *Synechocystis* sp. The increase of cell concentration to 3 × 10^6^ cells·mL^−1^ resulted in the capture of 80% of cells for *C. reinhardtii*; however, a further increase of the cell concentrations resulted in no significant increase of the percentage of chained cells (Figure S1a). The continuous rise of the percentage of the cells captured in chains was found for *C. meneghiniana*, reaching almost 100% of cells in chains at 8 × 10^6^ cells·mL^−1^ (Figure S1b). For *Synechocystis* sp., a 10× higher concentration (50 × 10^6^ cells·mL^−1^) was necessary to obtain inclusion of about 10% of cells in chains (Figure S1c). As the distance between the cells enters in the calculation of the DEP chaining force (Equation (1)) through the coefficient C [18,19], the size, as well as the concentration of the cells influence the distance between the cells and indirectly the chaining efficiency, as previously observed with the increase of the assembly rate by increasing the yeast cell concentration [19]. The small cell size (<2 µm) of *Synechocystis* sp. could explain the necessity to increase their concentration compared to the two other phytoplankton species (*C. reinhardtii* and *C. meneghiniana*) and thus to decrease the distance between these weakly polarizable cells.

#### 3.1.3. Effect of AC Field Duration on Chaining Efficiency

**Figure 3:**
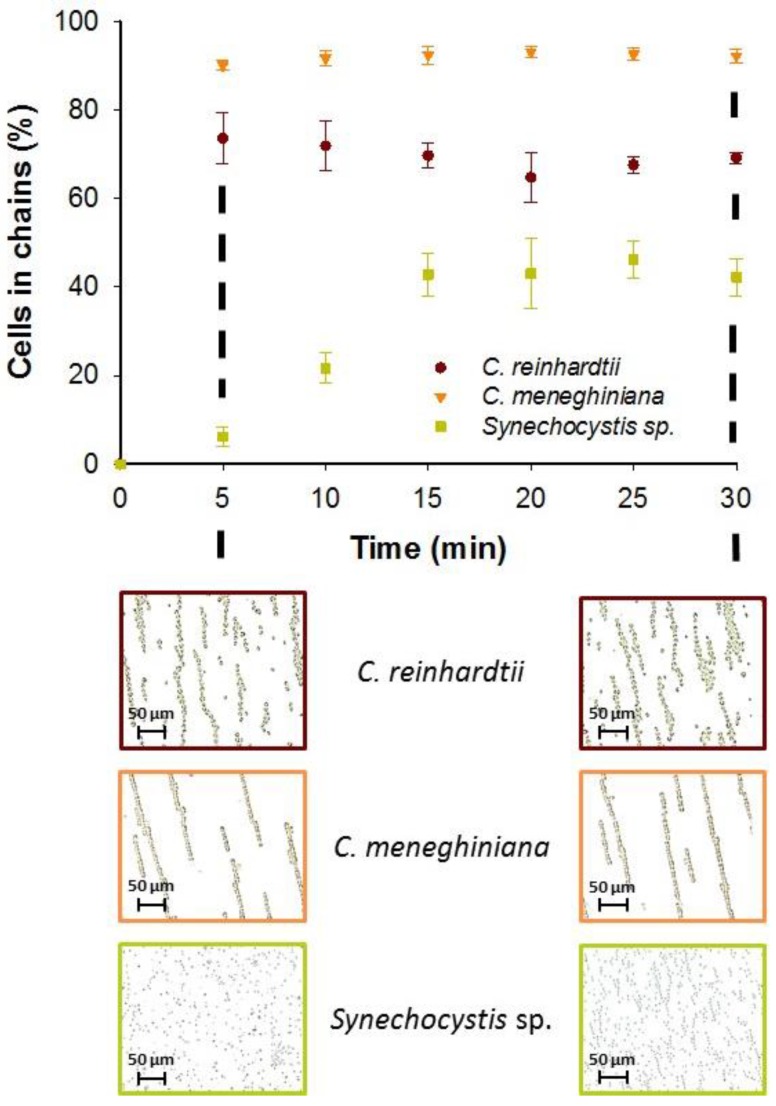
Effect of AC field duration on the chaining efficiency at 25 V·mm^−1^ and 500 kHz for *C. reinhardtii*, *C. meneghiniana* and *Synechocystis* sp. Micrographs obtained for each phytoplankton species after 5 min and 30 min of AC field application in Geneva Lake water. Initial concentration of *C. reinhardtii* and *C. meneghiniana*: 5 × 10^6^ cells·mL^−1^; *Synechocystis* sp.: 5 × 10^7^ cells·mL^−1^.

The AC field duration was also found to have a cell-specific effect on the efficiency of chaining. A collection time of 5 min resulted in a high percentage of *C. reinhardtii* (73.6% ± 5.8%) and *C. meneghiniana* (90.1% ± 1.2%) assembled in chains (Figure 3). However, a further increase of the time to 30 min has no effect on the percentage of cells captured in chains for both phytoplankton species. By contrast, the enhanced duration of AC field from 5 min and 15 min allowed a significant increase on the cell chaining efficiency of *Synechocystis* sp. from 6.2% ± 2.1% to 42.9% ± 4.8%, keeping steady until 30 min (Figure 3). The above observations were in agreement with the effective polarizability of the phytoplankton species. Rapid chaining formation was observed for the two phytoplankton species with low effective polarizability, while for the cells with high effective polarizability, such as *Synechocystis* sp., the duration of AC field application had a key role in the formation of chains using coplanar electrodes. Increasing collection time for cells with positive effective polarizability seemed to lead to an increase of the chaining efficiency.

Overall, these results confirm the cell-specific “dielectrophoretic phenotype” and show the importance of the optimization of field intensity, frequency and duration for each cell type in order to achieve the best collection conditions.

#### 3.1.4. Effect of the AC Field on the Cell Viability

No DEP-induced damages to the membrane permeability for the studied phytoplankton species was observed at 5 min of AC field duration at 25 V·mm^−1^ and 500 kHz (Figure S2). However, for the same combination of AC field intensity and frequency, a longer AC field duration of 30 min induced a shift in the PI fluorescence intensity for all phytoplankton species, showing significant membrane permeability damages. The membrane of *C. reinhardtii* seemed to be more impacted by the AC field after 30 min than the other two phytoplankton species, as their FCM cytograms showed a higher proportion of affected cells (Figure S2a) compared to *C. meneghiniana* and *Synechocystis* sp. (Figure S2b). Previous studies showed that the electric field could induce damage to yeast cells or bacteria with a long time AC field application [28,41]. For example, the number of viable yeast cells was reduced by 56.8% to 89.7% with a DEP treatment longer than 4 h [28], and trapping of genetically-modified *E. coli* was only workable for an AC field application of less than 10 min [41]. These results demonstrate the importance of the careful selection of the AC field duration to avoid damage of cell membrane integrity. Taking into account all of these results, an AC field duration of 5 min was preferred, since it allows one to achieve high chaining efficiency for *C. reinhardtii* and *C. meneghiniana*, while avoiding membrane damage.

### 3.2. Collection and “Pearl” Chain Formation by Artificial Communities

In natural waters, phytoplankton is composed of different species at different concentrations. In this section, mixtures containing equal concentrations of *C. reinhardtii*, *C. meneghiniana* or *Synechocystis* sp. and mixtures of *C. reinhardtii* or *C. meneghiniana* and 10-fold excess of *Synechocystis* sp. were used to evaluate the AC DEP-driven collection and chaining of the simple artificial community of phytoplankton species.

#### 3.2.1. Chaining of Binary Artificial Communities in Equal Concentration

**Figure 4:**
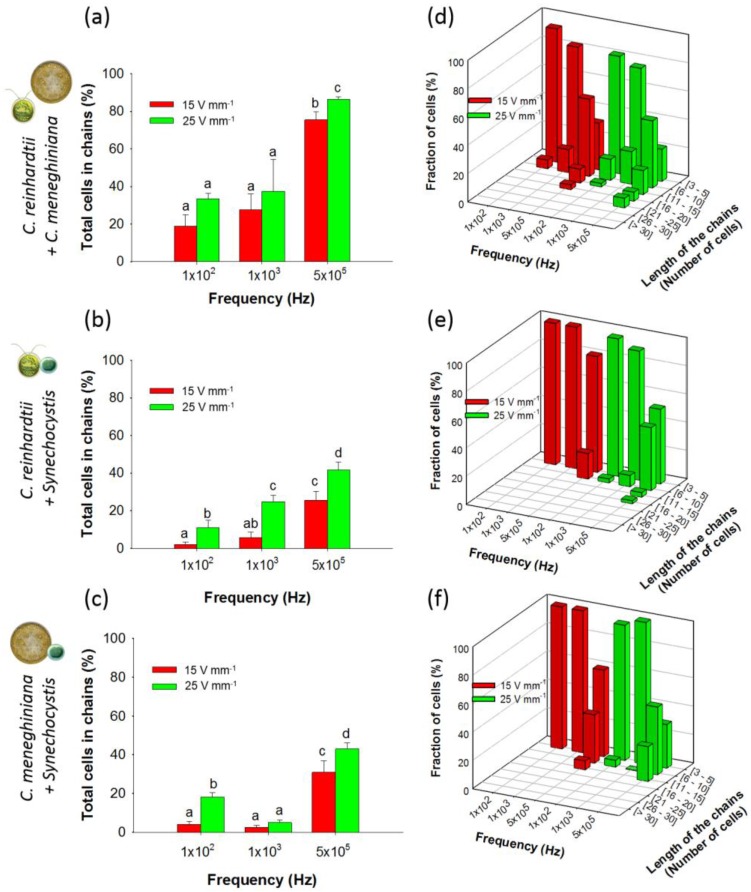
Chaining efficiency and length of mixed chains (1:1) at 15 and 25 V·mm^−1^ and at 100 Hz, 1 kHz and 500 kHz after 5 min of AC field application. Histograms represent the total percentage of cells in chains for: (**a**) *C. reinhardtii* + *C. meneghiniana*; (**b**) *C. reinhardtii* + *Synechocystis* sp.; and (**c**) *C. meneghiniana* + *Synechocystis* sp.; 3D histograms represent the fraction of cells in mixed chains of different lengths for: (**d**) *C. reinhardtii* + *C. meneghiniana*; (**e**) *C. reinhardtii* + *Synechocystis* sp.; and (**f**) *C. meneghiniana* + *Synechocystis* sp.; Different letters indicate significant differences between the measurements obtained by one-way ANOVA and the Student–Neumann–Keuls method (*p* < 0.05). Cell concentrations: 2.5 × 10^6^ cells·mL^−1^.

No significant differences in the chaining efficiency between 100 Hz and 1 kHz at 15 and 25 V·mm^−1^ were observed for artificial communities containing equal concentrations of *C. reinhardtii* and *C. meneghiniana* (Figure 4a).

Indeed, from 100 Hz to 1 kHz at both field intensities, chaining efficiency was always lower than 40% (Figure 4a). However, at a much higher frequency of 500 kHz, the chaining increased, substantially reaching 86.3% ± 1.5% at 25 V·mm^−1^ (Figure 4a). The length of the chains formed in the mixture containing *C. reinhardtii* and *C. meneghiniana* (Figure 4d) was comparable with those found for the individual species (Figure 2a,b). Furthermore, a higher number of long chains was observed for *C. reinhardtii* in presence of *C. meneghiniana* as compared with *C. reinhardtii* alone.

By contrast, adding *Synechocystis* sp. to *C. reinhardtii* or *C. meneghiniana* resulted in low chaining efficiencies at frequencies below 100 kHz at both AC field intensities (Figure 4a,b). An increase of the frequencies augmented the chaining efficiency to 45% for the community containing equivalent proportions of cyanobacteria and diatom or cyanobacteria and green alga at 500 kHz and 25 V·mm^−1^ (Figure 4a,b). However, the chains formed in such mixtures of *Synechocystis* sp. and *C. reinhardtii* or *C. meneghiniana* were shorter (Figure 4e,f).

Mixing *C. meneghiniana* and *C. reinhardtii* showed an increase of the percentage of *C. reinhardtii* in chains compared with the results obtained for *C. reinhardtii* alone, while a comparable percentage of *C. meneghiniana* in chains was obtained except at low frequencies (Figure S3). At 1 kHz, most of the chains were formed by *C. reinhardtii* cells (Figure S4a), which is consistent with the low chaining efficiency obtained for *C. meneghiniana* alone at this frequency (Figure 1b). However, for the other combination of AC field parameters, the chains were formed by the same proportion of *C. meneghiniana* and *C. reinhardtii* (Figure S4a). When mixed with *Synechocystis* sp., a decrease of the percentage of *C. reinhardtii* or *C. meneghiniana* in chains was observed (Figure S3c), and the chains were only formed by *C. reinhardtii* or *C. meneghiniana* cells (Figure S4b).

The above results showed that the presence of *C. meneghiniana* in the suspension increased the capability of *C. reinhardtii* to form chains at low frequencies and AC field intensities, while the presence of *Synechocystis* sp. decreased the capacity of *C. reinhardtii* and *C. meneghiniana* to form chains at all tested frequencies and AC field intensities. For the mixture of *C. reinhardtii* and *C. meneghiniana*, no significant changes in chaining were observed. Because of their similar polarizability, *C. reinhardtii* and *C. meneghiniana* aligned together in the direction of the electric field in the tested frequency range. Despite the difference in polarizability between *Synechocystis* sp. and *C. reinhardtii* or *C. meneghiniana*, no alternating chains were formed for mixtures containing *Synechocystis* sp., as previously observed with mixtures of yeast cells and polystyrene beads of lower and higher polarizability than the media using 2-mm gap coplanar copper electrodes [22]. The lack of *Synechocystis* sp. chaining, in parallel or in the transversal direction to the AC field, could be due to its low polarizability compared to the two other phytoplankton species, as well as a low cell density in *Synechocystis* sp., as cells’ concentration and size are important parameters affecting in the chaining efficiency, as mentioned above.

#### 3.2.2. Chaining of Binary Artificial Communities Containing an Excess of Cyanobacteria

**Figure 5:**
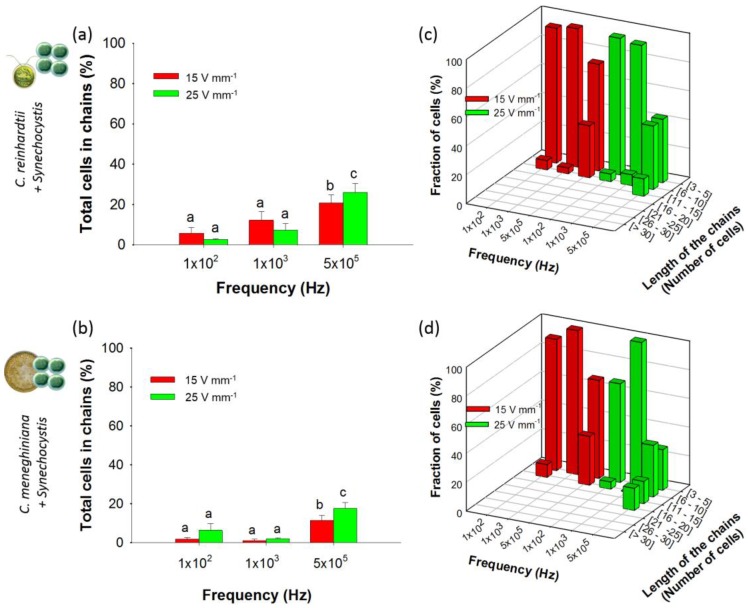
Chaining efficiency and structure of mixed chains (1:10) at 15 and 25 V·mm^−1^ and at 100 Hz, 1 kHz and 500 kHz after 5 min of AC field application. Histograms represent the total percentage of cells in chains for: (**a**) *C. reinhardtii* + *Synechocystis* sp.; and (**b**) *C. meneghiniana* + *Synechocystis* sp. 3D histograms represent the fraction of cells in mixed chains of different lengths for: (**c**) *C. reinhardtii* + *Synechocystis* sp.; and (**d**) *C. meneghiniana* + *Synechocystis* sp. Different letters indicate significant differences between the measurements obtained by one-way ANOVA and the Student–Neumann–Keuls method (*p* < 0.05). Concentrations of *C. reinhardtii* and *C. meneghiniana*: 2.5 × 10^6^ cells·mL^−1^; *Synechocystis* sp. concentration: 2.5 × 10^7^ cells·mL^−1^.

Suspensions with cyanobacteria excess were tested in this section to simulate episodically cyanobacteria blooms occurring in natural freshwaters. Under a 10-fold excess of cyanobacteria (2.5 × 10^6^ cells·mL^−1^ of *C. reinhardtii* or *C. meneghiniana* and 2.5 × 10^7^ cells·mL^−1^ of *Synechocystis* sp.), lower chaining efficiency at all frequencies and AC field intensities (Figure 5a,b) was observed as compared with the results found for artificial communities containing equal concentrations of two phytoplankton species. While previously, the chaining efficiencies at 25 V·mm^−1^ and 500 kHz reached 45% with 1:1 community containing *Synechocystis* sp. (Figure 4b,c), here, the chaining efficiencies was found to be only 26.1% ± 4.3% for *C. reinhardtii* and 17.5% ± 3.1% for *C. meneghiniana* in the presence of 2.5 × 10^7^ cells·mL^−1^ of *Synechocystis* sp. (Figure 5a,b). Under comparable DEP conditions, *C. reinhardtii* and *Synechocystis* sp. formed shorter chains at high AC field intensity and high frequency than *C. reinhardtii* alone (Figure 5c). Identical conclusions were made with the artificial community consisting of *C. meneghiniana* and *Synechocystis* sp. (Figure 5d). A decrease of the percentages of *C. reinhardtii* and *C. meneghiniana* in chains in the community containing cyanobacteria was observed (Figure S5a,c) compared to each phytoplankton alone, while the percentage of *Synechocystis* sp. increased when mixed with both *C. reinhardtii* and *C. meneghiniana* (Figure S5b,d). At most frequencies and AC field intensities, chains of phytoplankton mixtures containing *Synechocystis* sp. were only formed by *C. reinhardtii*, except at 500 kHz (Figure S4d), or by *C. meneghiniana*, except for 100 Hz and 500 kHz at 25 V·mm^−1^ (Figure S4e).

Overall, increasing the proportion of *Synechocystis* sp. in the artificial communities resulted in lowering the chain formation by *C. reinhardtii* and *C. meneghiniana*. Furthermore, under these conditions, *Synechocystis* sp. collection and chaining were observed when most of the cells of cyanobacterium were incorporated into the chains of green alga or diatom. Although we are not aware of previous studies of DEP of live cells mixtures that can be used for direct comparison, the findings of this study are consistent with the previous reports showing that small latex particles become dielectrophoretically-trapped between larger ones [24,42]. Primary chains of large particles were rapidly formed when the small particles were pulled in by DEP into the high-field intensity regions created by the pairs of large particles [36]. Moreover, it was shown that permanent chains of small and large particles of opposite charges only occurred when the small particles were in excess in the suspension [42]. The lack of alternating chains of *Synechocystis* sp., as observed in mixtures of particles with opposite polarizabilities [22], was attributed to the insufficient concentration or polarizability in *Synechocystis* sp. However, no alternating chains of *Synechocystis* sp. were observed even when this species was in excess in mixtures, which seemed to be due to its low polarizability. The results demonstrate how DEP-driven capturing and chaining under realistic conditions could be done while accounting for possible interactions between phytoplankton species existing in aquatic environments. These results highlight the possibility to capture selectively the phytoplankton species and to develop future biosensors based on the artificial communities better targeted to complex environmentally-relevant phytoplankton systems. By using various electrode configurations, DEP manipulation could allow one: (i) to discriminate different phytoplankton species depending on their effective polarizability and to enable their manipulation, such as specific collection or separation in freshwater; and (ii) to immobilize the cells in 1D or 2D arrays. Indeed, most of the present tools for water quality assessment are based on biosensors developed with only one phytoplankton species [7], whereas phytoplankton communities are composed of a large number of different microorganisms [1]. Future DEP-based biosensors could make use of the opportunity to form a 2D array of mixed phytoplankton species from freshwater, while allowing fluorescence microscopic observations, as we previously demonstrated with *C. reinhardtii* [17].

## 4. Conclusions

This study compares the DEP behavior of representative phytoplankton species alone and in mixture in order to evaluate the capability of DEP-driven selective collection and chaining on chip. Experimental results revealed different dielectrophoretic behavior for cyanobacteria and green alga or diatom in terms of the percentage of cells in chains and the length of the chains. Chaining efficiency and the length of chains of *C. reinhardtii* increased from 100 Hz to 500 kHz at all field intensities; *C. meneghiniana* showed a decrease of chaining efficiency from 100 Hz to 1 kHz followed by a significant increase of both chaining efficiency and chains length from 1 kHz to 500 kHz; while *Synechocystis* sp. showed low chaining efficiency at all frequency and field intensity combinations. The DEP behavior of these phytoplankton species was in agreement with the calculated values of the effective polarizability for these cells. Higher percentage of cells in chains and longer chains were observed for simple artificial communities containing *C. reinhardtii* and *C. meneghiniana*, while shorter chains and a lower percentage of cells in chains were obtained in all mixtures containing *Synechocystis* sp.

The results with individual cells and their mixtures representing artificial phytoplankton communities demonstrated the potential of DEP to manipulate representative phytoplankton species according to their DEP “phenotype” determined by their effective polarizability. In the future, we foresee the use of similar principles in new generations of devices for precise characterization of algal and microbial species in water. Separation or selective collection of the phytoplankton species from their natural environment could be conveniently achieved due to the differences in their polarizability. The precise control of frequency and fluid flows could allow continuous, on-line, cell species separation, detection and quantification for long-term monitoring of environmental water quality.

## Figures and Tables

**Table 1:** Numerical parameters used to calculate the effective polarizability of green microalga *C. reinhardtii*, cyanobacterium *Synechocystis* sp. and diatom *C. meneghiniana.*

	Symbol	Value			Units
		*C. reinhardtii*	*Synechocystis* sp.	*C. meneghiniana*	
Relative cytoplasm dielectric constant	*Ɛ*_2_	1.33 × 10^−9^ ^a^	5.40 × 10^−10^ ^b^	1.33 × 10^−9^ ^a^	C^2^·J^−1^·m^−1^
Cytoplasm conductivity	*σ*_2_	8.00 × 10^−3^ ^a^	1.90 × 10^−1^ ^b^	8.00 × 10^−3^ ^a^	S·m^−1^
Membrane capacitance	*c_m_*	1.42 × 10^−2^ ^a^	5.45 × 10^−2^ ^b^	1.42 × 10^−2^ ^a^	F·m^−2^
Relative cell wall dielectric constant	*Ɛ*_1_	6.20 × 10^−10^ ^a^	5.31 × 10^−10^ ^b^	3.45 × 10^−11^ ^c^	C^2^·J^−1^·m^−1^
Cell wall conductivity	*σ*_1_	5.00 × 10^−2^ ^a^	6.80 × 10^−1^ ^b^	1.00 × 10^−20^ ^c^	S·m^−1^
Inner radius	*R*	6.00 × 10^−6^ ^a^	1.83 × 10^−6^ ^b^	8.36 × 10^−6^ ^a^	m
Outer radius ^d^	*R*_0_	6.50 × 10^−6^	1.96 × 10^−6^	8.86 × 10^−6^	m
Zeta potential ^d^	*ζ*	−2.03 × 10^−2^	−1.82 × 10^−2^	−1.49 × 10^−2^	V
Double layer thickness ^e^	Δ	3.00 × 10^−9^	3.00 × 10^−9^	3.00 × 10^−9^	m
Debye length ^e^	*κ*^−1^	9.60 × 10^−9^	9.60 × 10^−9^	9.60 × 10^−9^	m
Relative medium dielectric constant ^e^	*Ɛ_m_*	7.08 × 10^−10^	7.08 × 10^−10^	7.08 × 10^−10^	C^2^·J^−1^·m^−1^
Medium conductivity ^d^	*σ_m_*	3.20 × 10^−1^	3.20 × 10^−1^	3.20 × 10^−1^	S·m^−1^
Angular frequency ^e^	*ω*	variable	variable	variable	Rad·s^−1^
Elementary charge ^e^	*e*	1.60 × 10^−19^	1.60 × 10^−19^	1.60 × 10^−19^	C
Boltzmann constant ^e^	*k*	1.38 × 10^−23^	1.38 × 10^−23^	1.38 × 10^−23^	J·K^−1^
Absolute temperature ^e^	*T*	298	298	298	K

^a^ Values of *C. vulgaris* taken from [30]; ^b^ values of *Escherichia coli* taken from [31]; ^c^ values of general properties of SiO_2_ taken from [32]; ^d^ values measured by Zetasizer Nano ZS, Malvern, this work; ^e^ Values taken from [19].

## Supplementary Materials

The following are available online at www.mdpi.com/2079-6374/7/1/4/s1: Table S1: Major physico-chemical parameters of Geneva Lake water; Figure S1: Effect of cell concentration on the chaining efficiency at 25 V·mm^−1^ and 500 kHz; Figure S2: Effect of AC-field application on membrane integrity using propidium iodide; Figure S3: Chaining efficiency of individual phytoplankton species in equal proportion mixtures compared to results obtained when alone in freshwater; Figure S4: Composition of mixed chains (1:1 and 1:10) for two voltages, 15 and 25 V·mm^−1^; Figure S5: Chaining efficiency of individual phytoplankton species in mixtures with predominant concentration of *Synechocystis* sp. compared to chaining efficiency obtained when alone in freshwater.
