# Supplementary material for: Alternating Current-Dielectrophoresis Collection and Chaining of Phytoplankton on Chip: Comparison of Individual Species and Artificial Communities
Source: Biosensors (Basel). 2017 Jan 5;7(1):4. doi: 10.3390/bios7010004 (PMC5371777; doi:10.3390/bios7010004)
Supplement: Supplementary file 1 [file biosensors-07-00004-s001.pdf]

Supplementary Materials: Alternating Current-Dielectrophoresis Collection and Chaining of Phytoplankton on Chip: Comparison of Individual Species and Artificial Communities

Coralie Siebman, Orlin D. Velev and Vera I. Slaveykova

Cell Cultures and Test Medium

Table S1. Major physico-chemical parameters of Geneva Lake freshwater.

Geneva Lake	
Conductivity (μS·cm^{-1})	320.0 ± 3.5
pH	8.2
Ca^{2+} (×10^{-3} M)	1.10 ± 0.05
Mg^{2+} (×10^{-3} M)	0.25 ± 0.02
Na^{+} (×10^{-3} M)	0.27 ± 0.01
K^{+} (×10^{-6} M)	39.0 ± 9.0
DOC (mg·L^{-1})	1.5 ± 0.1
Cl^{-} (×10^{-3} M)	0.25 ± 0.02
NO^{3-} (×10^{-3} M)	35
SO_4^{2-} (×10^{-3} M)	0.51 ± 0.02

DOC: Dissolved organic carbon.

Measurements of major anions and cations concentration in Geneva Lake freshwater were carried out by ion chromatography. The dissolved organic carbon concentrations were determined by TOC analyser. The values are average of 3 replicates.

Effect of Cell Concentrations on Chaining Efficiency

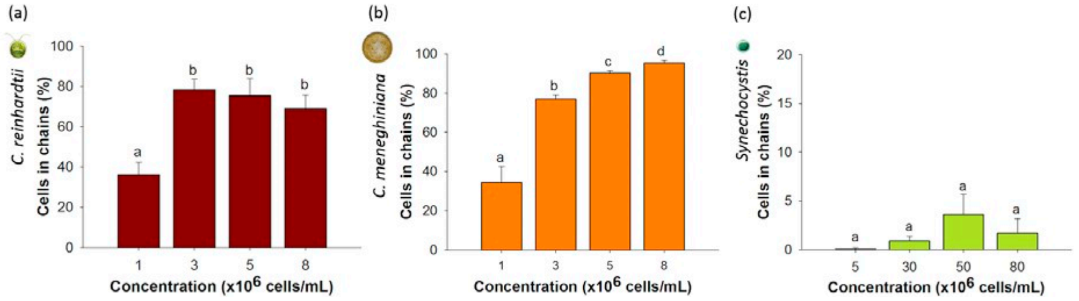

Figure S1. Effect of cell concentration on the chaining efficiency at 25 V·mm^{-1} and 500 KHz for (**a**) *C. reinhardtii*; (**b**) *C. meneghiniana*; and (**c**) *Synechocystis* sp. in Geneva Lake water. The different letters (from (**a**) to (**d**)) indicate statistically significant differences between the measurement ($p < 0.05$).

Effect of AC Field Application on Membrane Permeability

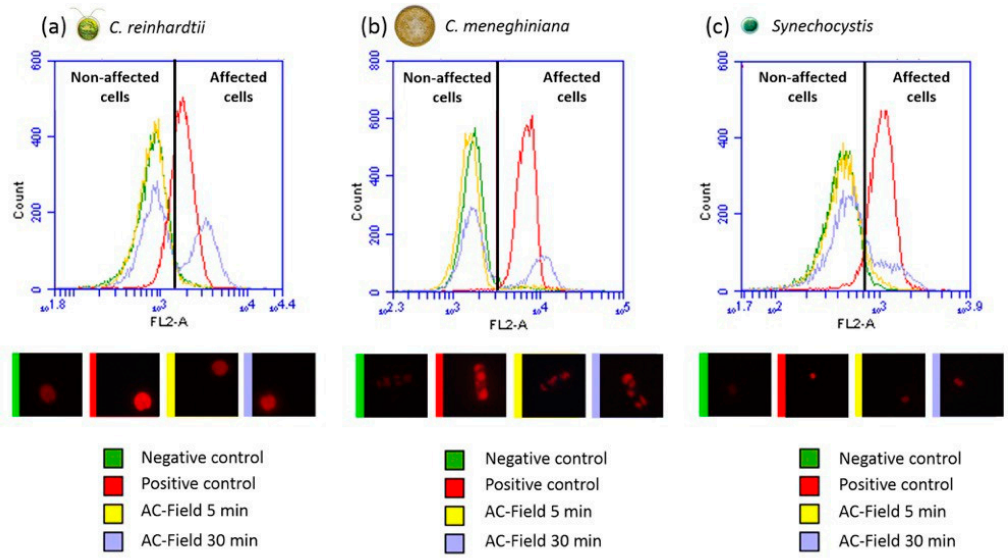

Figure S2. Effect of AC field application on membrane integrity using propidium iodide, after 5 min and 30 min of (**a**) *C. reinhardtii*; (**b**) *C. meneghiniana*; and (**c**) *Synechocystis* sp. FCM cytograms represent PI stained cells with AC field of 25 V·mm^{-1} and 500 KHz applied for 5 min and 30 min in Geneva Lake water.

Chaining of Binary Artificial Communities in Equal Concentration

The presence of *C. meneghiniana*, resulted in an increase of the percentage of *C. reinhardtii* in chains, which is particularly pronounced at 15 V·mm^{-1} at (Figure S3a), compared with the results obtained for *C. reinhardtii* alone (blue line in Figure S3a). The percentage of *C. meneghiniana* in chains when mixed with *C. reinhardtii* was comparable to the percentage of *C. meneghiniana* in chains alone except at low frequencies where the percentage of *C. meneghiniana* in chains decreased (Figure S3b). When mixed with *Synechocystis* sp., a decrease of the percentage of *C. reinhardtii* in chains was observed at 25 V·mm^{-1} (Figure S3) compared with the suspension of *C. reinhardtii* alone (blue line in Figure S3c). The percentage of *Synechocystis* sp. in chains did not increase when mixed with *C. reinhardtii or C. meneghiniana* at all tested parameters (Figure S3d,f). Finally, when mixed with *Synechocystis* sp., a decrease of the percentage of *C. meneghiniana* in chains was also observed at all frequencies and AC-field intensities (Figure S3e) compared with the suspension of *C. meneghiniana* alone (blue line in Figure S3e).

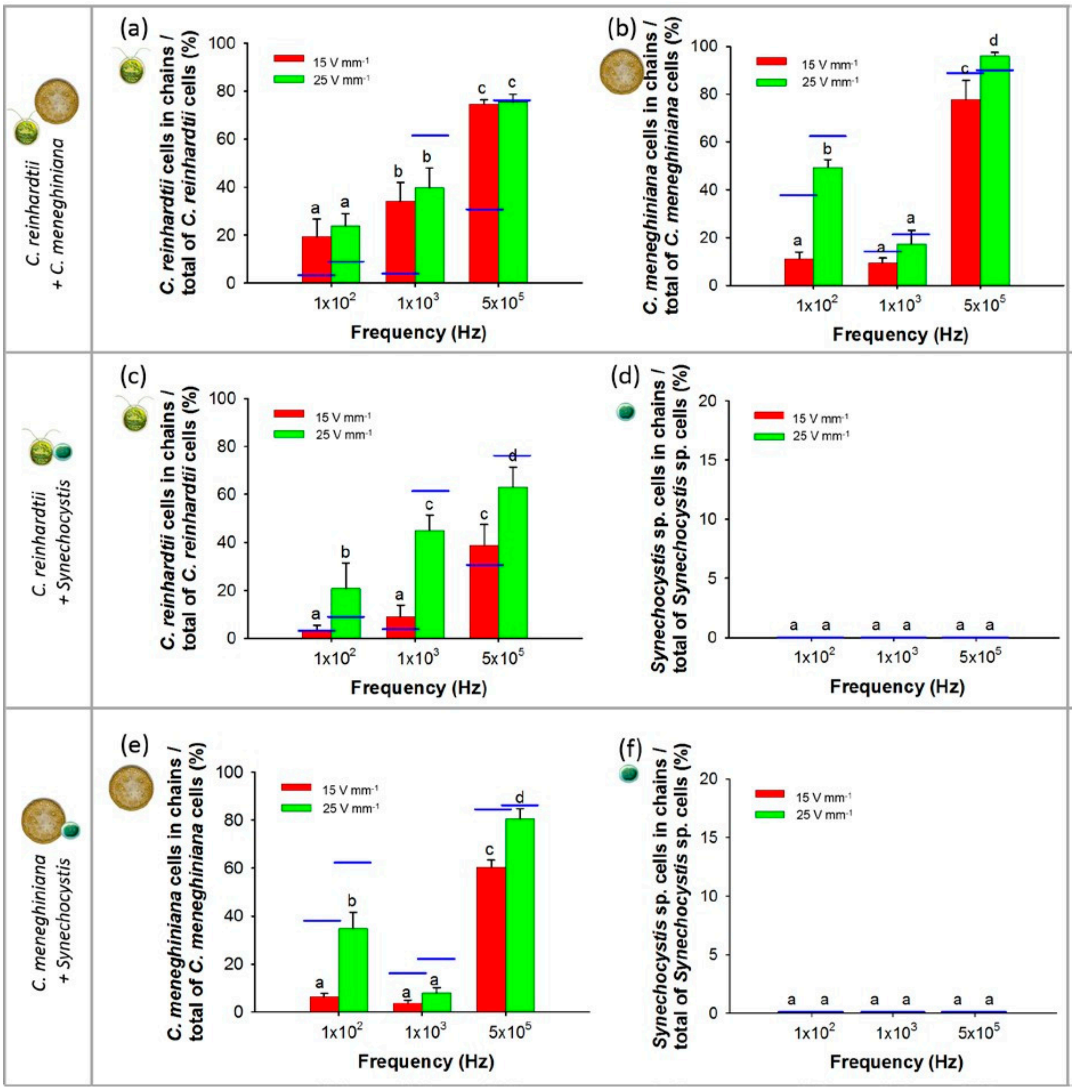

Figure S3. Chaining efficiency of individual phytoplankton species in equal proportion mixture (histogram) compared to results obtained when alone in freshwater (blue lines) at 15 and 25 V·mm^{-1} and at 100 Hz, 1 KHz and 500 KHz after 5 min of AC-field application. (**a**,**b**) Chaining efficiency of individual *C. reinhardtii* (**a**) and *C. meneghiniana* (**b**) artificial community; (**c**,**d**) Chaining efficiency of individual *C. reinhardtii* (**c**) and *Synechocystis* sp. (**d**) artificial community; (**e**,**f**) Chaining efficiency of individual *C. meneghiniana* (**e**) and *Synechocystis* sp. (**f**) when in artificial community. The different letters indicate statistically significant differences between the measurement ($p < 0.05$).

Fraction of Cells into the Chain

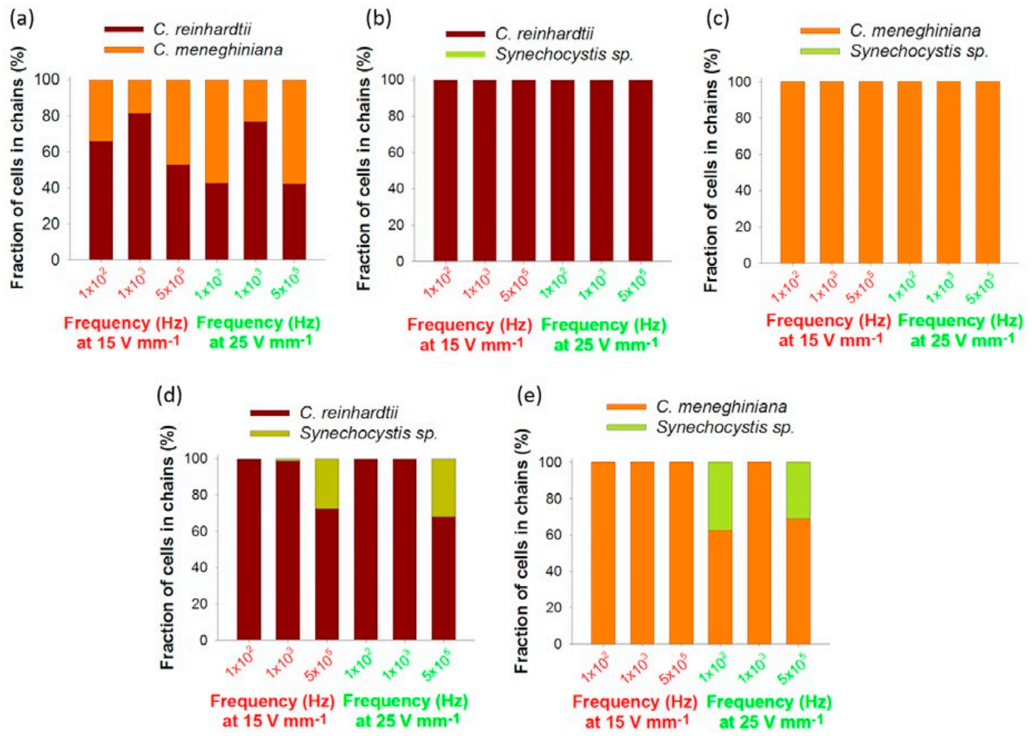

Figure S4. Composition of mixed chains (1:1 and 1:10) for two voltages, 15 and 25 V·mm^{-1}. Histograms represent the fraction of each cell into the formed chains for each mixture. (**a**) *C. reinhardtii* + *C. meneghiniana* (1:1); (**b**) *C. reinhardtii* + *Synechocystis* sp.; (**c**) *C. meneghiniana* + *Synechocystis* sp.; (**d**) *C. reinhardtii* + *Synechocystis* sp. (1:10); and (**e**) *C. meneghiniana* + *Synechocystis* sp. (**a**–**c**) Concentrations of cells at 2.5 × 10^6 cells·mL^{-1}. (**d**,**e**) Concentrations of *C. reinhardtii* and *C. meneghiniana* at 2.5 × 10^6 cells·mL^{-1} and concentration of *Synechocystis* sp. at 2.5 × 10^7 cells·mL^{-1}.

Chaining of Binary Artificial Communities Containing Excess of Cyanobacteria

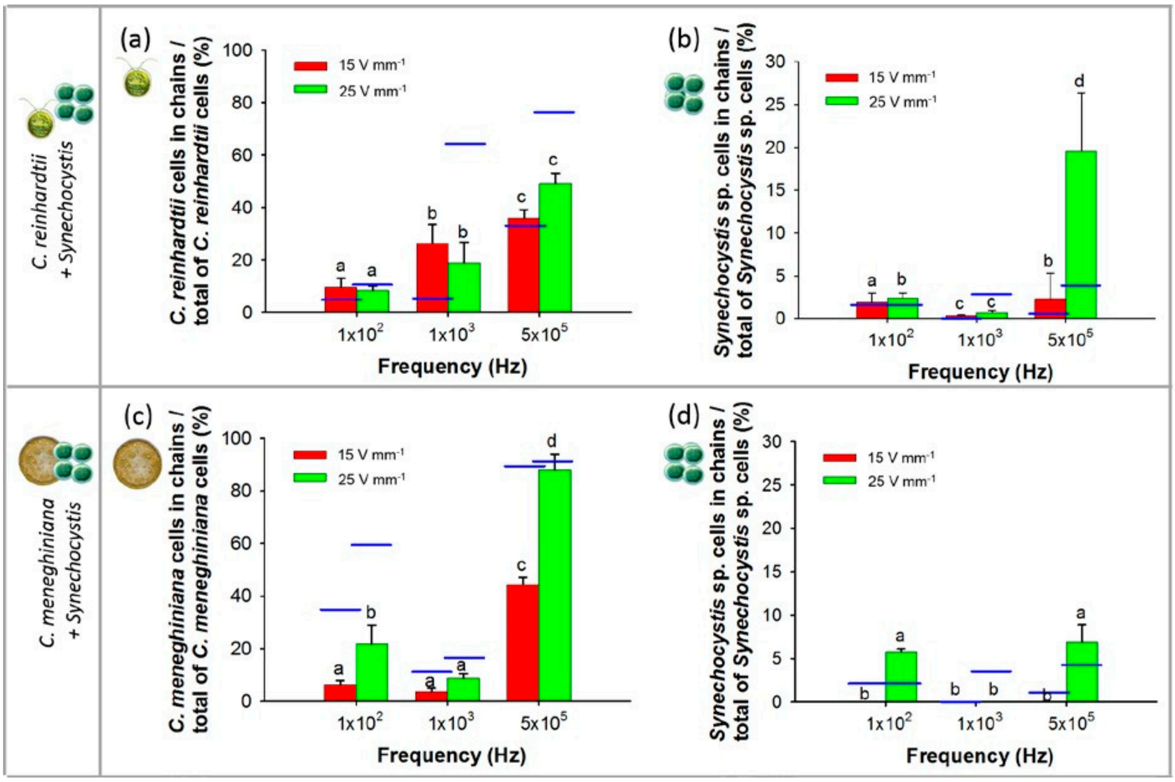

Figure S5. Chaining efficiency of individual phytoplankton species in mixture with predominant concentration of *Synechocystis* sp. (histogram) compared to chaining efficiency obtained when alone in freshwater (blue lines) at 15 and 25 V·mm^{-1} and at 100 Hz, 1 KHz and 500 KHz after 5 min of AC-field application. (**a**,**b**) Chaining efficiency of individual *C. reinhardtii* (**a**) and *Synechocystis* sp. (**b**) artificial community; (**c**,**d**) Chaining efficiency of individual *C. meneghiniana* (**c**) and *Synechocystis* sp. (**d**) artificial community. Concentrations of *C. reinhardtii* and *C. meneghiniana* were fixed at 2.5 × 10^{6} cells· mL^{-1} while *Synechocystis* sp. concentration was fixed at 2.5 × 10^{7} cells· mL^{-1}.

A decrease of the percentage of *C. reinhardtii* in chains in community containing cyanobacteria was observed at high AC-field intensity (Figure S5a) compared to *C. reinhardtii* alone (blue line in Figure S5a). The percentage of *Synechocystis* sp. increased at 15 V·mm^{-1} and 500 KHz to reach 20% of cells in chains when mixed with *C. reinhardtii* (Figure S5b) compared to 5% when alone (blue line in Figure S5b). As for *C. reinhardtii*, in presence of cyanobacteria, the percentage of *C. meneghiniana* in chains decreased especially at 15 V·mm^{-1} and at low frequencies for 25 V·mm^{-1} (Figure S5c) compared to *C. meneghiniana* chaining efficiencies when alone (blue line in Figure S5c). In contrary, the percentage of *Synechocystis* sp. increased at high AC field intensity in mixture containing *C. meneghiniana* (Figure S5d) compared to the level reached when *Synechocystis* sp. was alone (blue line in Figure S5d).
